# Cracking the Code: The Role of Peripheral Nervous System Signaling in Fracture Repair

**DOI:** 10.1007/s11914-023-00846-y

**Published:** 2024-01-18

**Authors:** Ashlyn J. Morris, Reginald S. Parker, Murad K. Nazzal, Roman M. Natoli, Jill C. Fehrenbacher, Melissa A. Kacena, Fletcher A. White

**Affiliations:** 1 Department of Orthopaedic Surgery, Indiana University School of Medicine, Indianapolis, IN USA; 2 Indiana Center for Musculoskeletal Health, Indiana University School of Medicine, Indianapolis, IN USA; 3 Department of Pharmacology and Toxicology, Indiana University School of Medicine, Indianapolis, IN USA; 4 Stark Neuroscience Research Institute, Indiana University School of Medicine, Indianapolis, IN USA; 5 Richard L. Roudebush VA Medical Center, Indianapolis, IN USA; 6 Department of Anesthesia, Indiana University School of Medicine, Indianapolis, IN USA

**Keywords:** Fracture healing, Neural regulation, Neuropeptides, Neurotrophins, Sympathetic nervous system, AI, Artificial intelligence, ChatGPT

## Abstract

**Purpose of Review:**

The traditionally understated role of neural regulation in fracture healing is gaining prominence, as recent findings underscore the peripheral nervous system’s critical contribution to bone repair. Indeed, it is becoming more evident that the nervous system modulates every stage of fracture healing, from the onset of inflammation to repair and eventual remodeling.

**Recent Findings:**

Essential to this process are neurotrophins and neuropeptides, such as substance P, calcitonin gene-related peptide, and neuropeptide Y. These molecules fulfill key roles in promoting osteogenesis, influencing inflammation, and mediating pain. The sympathetic nervous system also plays an important role in the healing process: while local sympathectomies may improve fracture healing, systemic sympathetic denervation impairs fracture healing. Furthermore, chronic activation of the sympathetic nervous system, often triggered by stress, is a potential impediment to effective fracture healing, marking an important area for further investigation.

**Summary:**

The potential to manipulate aspects of the nervous system offers promising therapeutic possibilities for improving outcomes in fracture healing. This review article is part of a series of multiple manuscripts designed to determine the utility of using artificial intelligence for writing scientific reviews.

## Introduction

This is one of many articles evaluating the utility of using AI to write scientific review articles on musculoskeletal topics [1]. The first draft of this review was written by ChatGPT 4.0 but was edited and carefully checked for accuracy resulting in a final manuscript which was significantly different from the original draft. Refer to this edition’s Comment paper for more information [2]. The process of fracture healing, recognized for its exceptional complexity, demands a sophisticated interplay among diverse cell types, signaling molecules, and tissue types [3]. Historically, the role of the nervous system in this dynamic biological concert has been undervalued. However, recent research trends have highlighted its crucial role in fracture healing [4]. As an entity known for its extensive reach and varied influence, the nervous system is now seen as a cardinal regulator that modulates each stage of healing, from the onset of inflammation to the final remodeling phase [5–7]. The necessity for a more profound understanding of this regulatory interplay is highlighted by continued challenges encountered in the clinical setting. Despite considerable advances in orthopedic care, complications such as fracture nonunion continue to impact a substantial number of patients [8]. Additionally, the escalating incidence of fractures, due to heightened activity levels in an aging population and an increase in osteoporosis-related fractures, underscores the need for the development of more efficacious therapeutic strategies [9, 10].

In this review, we aim to shed light on the diverse ways the nervous system influences fracture healing. We will provide a comprehensive analysis of the stages of healing, focusing on molecular and cellular mechanisms and highlighting the role of the nervous system at each step. The interplay of neuropeptides, the influences of the peripheral and sympathetic nervous system, and the intriguing role of neural regulation of stem cell function during fracture healing will be discussed. Moreover, we will investigate the therapeutic potential of targeting aspects of the nervous system to enhance fracture healing. This approach represents a promising frontier for improving patient outcomes and advancing fracture management strategies.

## The Neurobiology of Fracture Healing

Bone fractures, which may arise from a plethora of situations including trauma, osteoporotic pathologies, or high-impact activities, initiate a complex cascade of healing events within the human body [9, 11]. As our understanding of this process evolves, it is clear that the human body’s capacity to mend bone tissue and reinstate its function is nothing short of extraordinary. Healing after a fracture proceeds through three distinct yet interconnected stages, namely, the reactive phase, the reparative phase, and the remodeling phase [11–13]. These sequential stages are intricately guided by the nervous system [14].

The initial reactive phase serves as the body’s immediate reaction to a fracture [13]. The disruption of blood vessels within the periosteum and bone marrow results in the formation of a hematoma, inciting an inflammatory response [15]. This response is marked by the mobilization of immune cells and the secretion of growth factors such as platelet-derived growth factor (PDGF) and transforming growth factor-beta (TGF-β), which are instrumental in the early healing response [16]. The nervous system exerts a significant influence during this phase, modulating the inflammatory response by releasing neurotransmitters and neuropeptides [17–20]. These substances harmonize the recruitment of immune cells, promote angiogenesis, and regulate blood flow to the fracture site [21–23].

The subsequent reparative phase emerges post-inflammation, marked by the formation of a soft callus chiefly constituted by cartilage and immature bone [12]. Here too, the nervous system’s influence remains paramount: it facilitates the differentiation of mesenchymal stem cells (MSCs) into osteoblasts and chondrocytes, cells that lay the foundation for new bone matrix formation [11]. Neuropeptides, such as substance P (SP) and calcitonin gene-related peptide (CGRP), govern the differentiation and proliferation of these bone-forming cells, enabling the transition from soft to hard callus [7, 24, 25].

The final remodeling phase signifies the conversion of woven bone into lamellar bone, reestablishing the original bone architecture [11]. The influence of the nervous system persists, primarily through its regulation of osteoclast activity and, thereby, bone resorption [26–28]. The transformation of the callus into mature bone is additionally modulated by neuropeptides, including CGRP, which encourage bone formation, ensuring a balance between osteoblastic and osteoclastic activity and allowing for lamellar bone deposition alongside callus resorption [25, 29].

Taken together, the neurobiology of fracture healing emphasizes a sophisticated dynamic between the nervous system and bone repair mechanisms. This critical contribution of the nervous system to successful bone regeneration sets the stage for our subsequent in-depth exploration of the role of individual neuropeptides and neurotrophins in the healing process.

## The Functions of Neuropeptides and Neurotrophins in Fracture Healing

Fracture healing is a complex process steered by a myriad of signaling molecules, including but not limited to neuropeptides [30]. Neuropeptides, small protein-like molecules, are essential communication tools for neurons in both the central and peripheral nervous systems [31, 32]. They have a vital role in modulating the course of fracture healing, orchestrating functions such as inflammation, angiogenesis, and cellular differentiation [33]. Neurotrophins, another class of small peptide molecules, also play an important role following fractures by influencing bone formation, promoting axonal regrowth and guidance, and by enhancing pain sensitivity during the healing process [18, 34•]. This section further explores their specific roles, starting with an overview of the contributions of the peripheral nervous system (PNS) to fracture healing.

### The Role of the Peripheral Nervous System in Fracture Healing

A vast network of nerves innervates all aspects of bone, including the bone marrow, trabecular bone, cortical bone, and the periosteum [35. 36•, 37]. Resection of the sciatic nerve, which contains sensory, sympathetic, and motor fibers, has historically been a model used to investigate the overall impacts of the PNS on fracture healing [38–42]. In a string of experiments, sciatic denervation led to more rapid callus bridging and the formation of larger calluses for tibial fractures [38–40, 42]. However, this larger callus was not mechanically stronger, with researchers suggesting that a lack of guidance from the PNS during the healing process led to defective callus organization [39, 42]. An important caveat to these findings is that resection of the sciatic nerve has been shown to be insufficient in producing a completely denervated fracture as researchers have found nerve fibers regenerating in the bone marrow, callus, and periosteum following sciatic nerve resection [40, 42]. Nonetheless, these experiments did show a relationship between the PNS and fracture healing, which set the stage for future experiments that more clearly delineated the impact of individual nerve types on fracture healing.

Additional research has elucidated the fact that neurons play a decisive role in fracture healing by releasing an array of neuropeptides and neurotrophins that regulate numerous aspects of the healing process, including inflammation, pain, and the stimulation of bone cell proliferation and differentiation [6, 17, 24, 43–46]. In addition to the release of these small molecules, nerve axons also undergo a noticeable, controlled sprouting at the fracture site to better support the healing process such that normal function and sensation of the affected part of the body can be restored [47–50]. As healing concludes, these nerve axons are pruned back. In the case of fracture nonunion though, researchers have demonstrated a much more prolific growth of both sensory and sympathetic nerve axons at the fracture site, with significant associated pain behaviors observed in the affected mice [50].

Schwann cells, the predominant glial cells of the PNS, also have an intriguing role in the fracture healing process as a component of the microenvironment that promotes the transformation of osteoprogenitor cells into osteoblasts following bone trauma [51–53]. In addition, Schwann cells secrete vascular endothelial growth factor (VEGF), a potent promoter of angiogenesis, during bone healing [51, 53].

### Neuropeptide: Substance P

SP, a neuropeptide primarily released by the distal axons of primary afferent sensory neurons and inflammatory cells including macrophages and lymphocytes, demonstrates a diverse and integral role in fracture healing, encompassing functions such as promoting inflammation, modulating osteoclast and osteoblast activity, and transmitting pain [24, 43, 54–59]. Although many of the studies implicating SP’s involvement in fracture healing have been performed in animals, plasma levels of SP have been found to be elevated for up to 48 h in humans following femoral neck fractures [60].

During the reactive phase of fracture healing, SP acts as a potent catalyst of neurogenic inflammation, a physiological response typified by vasodilation, increased vascular permeability, and chemotaxis of monocytes [54, 56, 59, 61, 62]. Evidence for SP’s vasodilatory effects comes from application of SP to arteries isolated from the cancellous bone of pigs, which elicited a transient relaxation of the arteries [21]. SP also fosters the mobilization of stromal cells from connective tissues, likely including bone marrow, to the site of injury [43]. This idea is supported by experiments in which SP was intravenously administered to uninjured mice, resulting in the mobilization of CD29 + stromal cells, including bone marrow stromal cells, to the peripheral blood [43].

SP also has important effects on osteoclastogenesis and osteoclastic activity. NK-1 receptors that bind SP are expressed by osteoclasts (as well as osteoblasts and bone marrow stem cells), and SP addition to a culture of osteoclast progenitor cells has been shown to increase osteoclastogenesis via activation of NF-κB [27, 63–65]. In addition, administration of SP to cultured osteoclasts led to increased bone resorption activity by the osteoclasts, while administration of an SP antagonist inhibited this bone resorption [28].

Furthermore, SP has been shown to stimulate the proliferation of osteoblasts and differentiation of chondrocytes, the primary cell types involved in callus formation during the reparative phase of fracture healing [7, 24, 54]. In an in vitro study of rat calvarial osteoblastic cells, an observable increase in the size of the mineralized nodules produced was observed when the cells were exposed to SP [24]. Further, a decreased number of osteoblasts and chondrocytes were detected in the fracture callus of SP-deficient mice, highlighting the neuropeptide’s influence on cellular proliferation [7].

Other studies have begun to clarify SP’s seemingly contradictory involvement in both bone formation and bone resorption during fracture healing [19]. Specifically, in an angulated fracture model, SP + peripheral nerve fibers were found in high concentrations on the concave loaded side of the fracture during bone regeneration, with the peak concentration of SP + nerve fibers corresponding to the areas of greatest bone formation. Later in the process of healing, during the remodeling phase, SP + nerve fibers were found on the convex unloaded side of the fracture where bone resorption was occurring. Thus, this experiment suggests a time-dependent role of SP during fracture healing, with the neuropeptide first stimulating bone formation during the reparative phase and then impacting bone resorption during the remodeling phase.

Finally, a complex role of SP is seen in the arena of pain transmission linked with bone fractures [66, 67]. Released by primary afferent sensory neurons in reaction to injurious stimuli, SP influences pain by binding to its receptor, neurokinin-1, present in both the central and peripheral nervous systems [33]. In mice deficient in SP, decreased nociceptive responses to moderate and severe noxious stimuli, including tail clipping and capsaicin injection, have been observed [66]. Notably, in orthopedic indications, such as hip osteoarthritis (OA), patients who are in pain have an increased density of nerve fibers containing substance P in the hip joint capsule and acetabular fossa, while non-OA controls (femoral head fracture) who experience no pain lacked local nerve fibers containing substance P [67]. Taken together, these findings underscore the intricate involvement of substance P in the sensory, inflammatory, and reparative elements of musculoskeletal regeneration.

### Neuropeptide: CGRP

CGRP, another pivotal neuropeptide, exhibits considerable influence on the process of fracture healing, a claim substantiated by numerous studies [47, 68••, 69, 70]. Similar to SP, elevated plasma levels of CGRP have been found in humans following femoral neck fractures [60]. Moreover, administration of gelatin microspheres containing CGRP improved the healing of a bone defect, with noted increased bone volume density, in an osteoporosis model [71]. Further, application of a CGRP-supplemented fibrin sealant during a partial patellectomy led to increased bone mineral composition during the healing process. The ultimate strength, stiffness, and failure load in the affected limb were all enhanced in these mice [72]. Conversely, mice deficient in CGRP have impaired fracture healing, as indicated by high rates of incomplete callus bridging, reduced callus volumes, decreased bone mass with a corresponding reduced number of osteoblasts, and decreased bone strength [68••, 73]. Additionally, injection of a CGRP inhibitor has been shown to impair fracture healing [74].

CGRP’s contribution to bone formation has been affirmed through its enhancement of osteoblast differentiation and inhibition of osteoclast activity [25, 73, 75, 76]. Treatment of bone marrow stromal cells in vitro with CGRP led to cellular proliferation, increased expression of osteoblastic genes including Runx2, and ultimately increased osteoblastic differentiation [29, 68••, 77]. Also, the administration of higher concentrations of CGRP to cultures of rat bone marrow cells led to the formation of larger numbers of bone colonies in a dose-dependent manner [25]. Further evidence for the role of CGRP in stimulating osteogenesis comes from studies showing a positive correlation between the areas of greatest bone formation during fracture healing and CGRP levels in the area [19].

In addition to its promotion of bone formation, CGRP also acts directly on osteoclasts to inhibit osteoclast-driven bone resorption [29, 78]. CGRP application has been shown to downregulate osteoclastic genes, including TRAP and cathepsin K, and CGRP also decreases the bone resorption activity of RANKL-induced bone marrow macrophages [29]. When examined alongside its promotion of osteoblastic differentiation and activity, these functions point to CGRP’s role in maintaining and increasing bone mass, which is important for successful fracture healing.

Furthermore, CGRP has been strongly implicated in pain regulation, particularly in increasing nociceptive transmission in both the peripheral and central nervous systems following injury [67, 79]. Experiments involving rats demonstrated an increased pain response, as measured through paw withdrawal threshold testing when CGRP was intrathecally administered, thereby establishing its integral role in nociception [80]. Further, von Frey tactile testing has illustrated that CGRP antagonists reverse the mechanical allodynia that is observed in mice following fracture [81]. Since hyperalgesia is also diminished by the simultaneous administration of CGRP with a PKA or PKC inhibitor, researchers have suggested CGRP nociceptive signaling is mediated via the PKA and PKC second messenger pathways [80]. Additionally, it has been demonstrated that administration of an IL-1 receptor antagonist alongside CGRP prevented mechanical allodynia in mice [82]. When considered alongside the fact that keratinocyte expression of IL-1 is normally upregulated following CGRP administration in a dose-dependent manner, these researchers suggested that CGRP induces hyperalgesia via enhancement of IL-1 expression. Overall, the wide-ranging impact of CGRP underscores its paramount significance in the neural regulation of the fracture healing process, reflecting its influences on cell differentiation and pain regulation.

### Neuropeptide: Neuropeptide Y

Neuropeptide Y (NPY), another crucial neuropeptide present in both sympathetic and primary afferent sensory neurons, has become a focal point of research into fracture healing due to its burgeoning role in the process [20, 48, 83]. Interestingly, though, studies of the impact of NPY on bone homeostasis have shown that NPY has an anti-anabolic effect on bone mass. NPY interacts directly with osteoblasts via the Y1 and Y2 receptors, and deletion of either of these receptors in mice led to an increase in osteoblastic activity with a corresponding increase in bone formation and bone mass [84]. Similarly, when osteoblasts were cultured with NPY, they exhibited decreases in markers of differentiation and in the extent of mineralization [85]. Additionally, a decrease in osteoid width and osteoblastic activity was observed upon NPY injection in mice [86]. Considered together, these findings suggest that NPY has a negative influence on bone homeostasis via its inhibition of osteoblastic activity.

However, in contrast to its role in bone homeostasis, NPY has a positive effect on bone healing following fracture. NPY’s significance has been demonstrated through studies that used NPY-deficient mice as models. Specifically, these studies revealed impairments in the earlier stages of fracture healing, as evidenced by decreased callus size, decreased callus strength, and delayed callus bridging, in mice with germline deletion of NPY [20]. Moreover, a study of humans experiencing craniocerebral injuries determined that elevated serum levels of NPY were associated with accelerated fracture repair times [83]. Further evidence comes from immunohistochemical analysis of angular fractures in rats, which found an increased concentration of NPY + fibers on the concave side of the fracture during the reactive phase [48]. In addition, a high concentration of NPY + nerve fibers was found on the convex side of the fracture between 21 and 56 days. Since this time period is correlated with that in which the convex side of the fracture callus was decreasing in size, this suggests that NPY has a hand in the remodeling phase of fracture healing in addition to its role during earlier phases [48].

Moreover, NPY plays a role in pain modulation, a crucial aspect of the body’s response to fractures. NPY’s effect on pain differs based on which of its receptors it binds, with activation of Y1 inhibiting pain and Y2 agonism potentially promoting pain [87]. Evidence for this has been documented in studies involving Y1 receptor knockout mice, in which the animals showed an escalated pain response and exhibited mechanical hypersensitivity [88]. Additionally, exogenous NPY administration increased latency to paw withdrawal from a heat source and reduced molecular markers of inflammatory pain, while administration of a Y1 antagonist inhibited these results [89–91]. Further, addition of either a Y1 agonist or synthetic NPY to slices of rat spinal cord dorsal horn inhibited the exocytosis of the nociceptive CGRP from capsaicin-sensitive centrally-projecting terminals in the dorsal horn, suggesting a possible mechanism through which NPY inhibits nociception [92]. These experiments suggest NPY’s potential in inhibiting pain transmission within the central nervous system via Y1 receptors.

In contrast to the relatively well-defined role of Y1 receptors, the impact of NPY on Y2 receptors in regard to pain is more controversial. Specifically, it was found that administration of a Y2 agonist increased CGRP release from rat trigeminal ganglia, suggesting that activation of Y2 receptors may lead to increased pain [87]. This idea is supported by the finding that Y2 antagonist administration inhibited NPY-induced mechanical allodynia [93]. However, other researchers have instead found that Y2 antagonists, like Y1 antagonists, inhibit the analgesic effect of NPY [90]. Additional studies are needed to better differentiate the scenarios in which NPY causes or relieves pain. In summary, the versatile role of NPY demonstrates its importance in the intricate neurobiological regulation of fracture healing, spanning from its influence on osteoblast activity to pain modulation.

### Neurotrophins

Neurotrophins comprise a class of proteins, including but not limited to nerve growth factor (NGF) and brain-derived neurotrophic factor (BDNF), which play a pivotal part in the healing process [18]. Studies using animal models lacking these factors have revealed impaired sensation and increased neurodegeneration, showcasing their crucial roles in nerve regeneration and neuronal survival [94, 95]. In addition to their impact on nerve regeneration, both NGF and BDNF have been found within fracture tissues, suggesting these neurotrophins also play important roles in bone regeneration and fracture healing [18, 96, 97]. Specifically, while expression of NGF is limited to the periosteum under normal conditions, following fracture, it is found around the fracture callus in marrow stromal cells, osteoprogenitor cells, osteoblasts, and osteocytes, with NGF mRNA levels reaching a peak 2 days after the fracture [18, 97]. Additionally, NGF has been shown to contribute to sensory and sympathetic nerve axon sprouting following peripheral nerve injury, and there is some evidence that NGF contributes to nerve sprouting following fracture as well [46, 98]. BDNF has been localized to osteoblastic and endothelial cells during the reactive and early reparative phases, suggesting a primary role in the earlier stages of fracture healing [18, 96].

A variety of experiments have implicated both BDNF and NGF in various aspects of bone formation and resorption during fracture healing. Indeed, BDNF and NGF are both known to stimulate osteoblast proliferation and differentiation [99]. BDNF has also been shown to increase release of RANKL from bone marrow stromal cells and thus have a role in osteoclastogenesis [100]. Also, increased cartilage differentiation and increased formation of osteoclasts were observed in NGF transgenic mice with induced tibial fractures [101].

Neurotrophic factors also influence MSCs during bone healing. NGF and BDNF promote MSC survival and differentiation, steering them toward becoming osteoblasts and chondrocytes, the bedrock units of bone and cartilage, respectively [102–104]. It is possible that these factors are also involved in guiding MSCs to fracture sites, a critical precursor step to callus formation [102]. Furthermore, these factors are a pro-survival factor in the balance between MSC proliferation and apoptosis—a delicate equilibrium vital for maintaining tissue homeostasis throughout the healing process [105].

Finally, neurotrophins have significant implications for pain modulation and sensitization. Studies have demonstrated that a decrease in NGF signaling due to application of anti-NGF antibodies following fracture correlates with reduced pain-related behaviors [106, 107]. Further, mice treated with anti-NGF therapy demonstrated increased activity following fracture, with the researchers suggesting that this finding was due to a decreased experience of pain in the treated mice [108].

In summary, neuropeptides and neurotrophins play important and often synergistic roles in fracture healing. For example, SP and CGRP are frequently colocalized within the same primary afferent sensory neurons, and it has been hypothesized that they are released together following injury [33]. Further, NGF likely plays a role in the recruitment of the nerve axons containing these neuropeptides and can function to upregulate the expression of both SP and CGRP [46, 109, 110]. Once released, both SP and CGRP play a role in bone formation by increasing osteoblast activity. Another interaction between neuropeptides occurs in the area of pain transmission, where NPY inhibits the release of CGRP from the spinal cord dorsal horn and thus diminishes nociception [92]. Ultimately, all of the small molecules discussed in this section, including SP, CGRP, NPY, NGF, and BDNF, work in concert to promote fracture healing and to impact pain transmission. The intricacies of their functions emphasize the complexity of the healing process and pave the way for the subsequent discussion of the sympathetic nervous system’s role in fracture healing.

## The Role of the Sympathetic Nervous System in Fracture Healing

### Impact of the Sympathetic Nervous System on Fracture Healing

Studies have reliably demonstrated that the sympathetic nervous system (SNS) innervates bone and that adrenergic receptors are present on both osteoblasts and osteoclasts [111–113]. Although additional research is still needed, the aggregate of the findings so far suggests that while local sympathetic denervation improves fracture healing, complete knockout of the SNS impairs fracture healing.

Experiments dating back to the mid-twentieth century have supported the idea that local sympathectomy increases blood flow to damaged tissues, potentially resulting in an increase in bone growth and an acceleration of fracture healing [114, 115]. For example, when these experimenters fractured the hind legs of 12 dogs after performing a lumbosacral sympathectomy on them, they determined that there was a marked enhancement of the healing in 11 of the cases [115]. More recent experiments have provided support for this idea. When the mandible of rats was fractured and a bone-borne distractor implanted to induce distraction osteogenesis (DO), rats who had simultaneously experienced a cervical sympathetic trunk transection exhibited increased bone mineral density and more continuous bone formation at 14 days when compared to those rats with intact cervical sympathetic trunks [116].

Building on this experiment, researchers again used a rat model of mandibular DO with a simultaneous cervical sympathetic trunk transection to better understand the influence of the SNS on mesenchymal stem cells (MSCs) [117]. Immunohistochemical staining for nestin, a marker for MSCs, demonstrated an increased number of MSCs in bone-forming areas in the sympathetically denervated rats when compared with controls whose MSCs largely remained within their perivascular stem cell niche. It was also shown that norepinephrine, the primary neurotransmitter released by the SNS, prevented the osteogenic differentiation of MSCs, while downregulation of the β3-adrenergic receptor (*ardb3*) mitigated this norepinephrine-dependent inhibition of differentiation [117]. Taken together, these results show that the SNS decreases the migration and differentiation of MSCs, suggesting again that the intact SNS may exert an inhibitory control on bone growth and healing.

Additional support for the idea that inhibition of the SNS improves fracture healing comes from studies that manipulated the interaction between sensory and sympathetic nerves. Prostaglandin E2, a known mediator of pain, is secreted by osteoblasts when bone density decreases [118••]. Evidence suggests that this substance acts on EP4 receptors present on primary afferent sensory nerve fibers to inhibit sympathetic tone and via this pathway stimulate an increase in bone density. Likely due to the inhibitory effect of sympathetic signaling on MSC activity, EP4 receptor knockout mice displayed decreased MSC differentiation to osteoblasts and diminished osteogenesis [119].

On the other hand, there is a growing amount of research insinuating that total elimination of the SNS diminishes aspects of fracture healing. Our understanding of the effects of total sympathectomy has been greatly enhanced through the strategic use of neurotoxins like 6-hydroxydopamine (6-OHDA) [17, 120]. This potent compound is known for its deleterious impact on peripheral sympathetic nerve fibers, culminating in the interrupted production of norepinephrine [17, 120]. For example, upon injecting 6-OHDA into a mouse model, the measured amount of mineralized bone was decreased and the structural integrity of bones was compromised in the time period following femoral fractures, thereby underlining the crucial role of the SNS in optimal bone recovery post-trauma [120]. Further, in a second study that examined the femoral fracture healing trajectory in mice devoid of sympathetic innervation due to 6-OHDA application, mice displayed decreased bone stability and a pronounced delay in bony callus development [17]. This provides additional evidence for the integral role of the SNS during bone repair. However, it is important to note that pharmacological drugs, like 6-OHDA, that were used in these studies may have poorly characterized impacts on the bone microenvironment that could also impact these results [121].

Finally, the SNS’s function may extend to pain modulation during fracture healing [7]. Following a fracture, several neurotransmitters, cytokines, and growth factors are released which cause the proliferation of new sympathetic fibers that augment the perception of pain during the healing trajectory [50, 122]. However, while some experiments have found evidence for a heightened threshold for touch sensitivity after fracture in completely sympathectomized mice, another experiment concluded that mice treated with 6-OHDA displayed no difference in withdrawal thresholds [7, 17, 123]. In light of these research findings, the role of the SNS in fracture healing is revealed as profoundly complex, encompassing not only the physical repair process but also potentially the modulation of pain perception.

### The Impact of Stress and Sympathetic Nervous System Activation on Fracture Healing

Chronic stress and its resultant chronic activation of the SNS are recognized as potential hindrances to the healing process [124, 125, 126••, 127]. Through the continuous release of norepinephrine, chronic stress induces a dysregulation of immune responses that can deleteriously affect fracture healing [126••, 128, 129]. Chronic stress can be induced in mice via the chronic subordinate colony housing paradigm, in which male mice are continuously exposed to a dominant male aggressor. In mice with fractures who are experiencing chronic stress, researchers have demonstrated reduced neoangiogenesis at the fracture site, decreased rates of chondrocyte-to-osteoblast transdifferentiation, and poor functional fracture healing outcomes when compared to controls [126••]. Additional evidence exists for the idea that chronic stress impairs endochondral ossification [127]. Further, an imbalanced immune response to fractures, characterized by increased neutrophils and decreased lymphocytes at the fracture site, was shown to be mediated by adrenergic signaling in chronically stressed mice [126••].

Chronic stress is also known to influence pain perception and may theoretically exacerbate pain experiences associated with fractures [130–132]. This has significant implications for patient comfort and recovery, particularly in instances of complex or slow-healing fractures. In the case of complex regional pain syndrome, lumbar sympathetic blocks have beneficial impacts on pain and functionality, suggesting a potential area for investigation for the treatment of chronic stress-induced pain in fracture patients [133, 134]. Further research is necessary to elucidate the relationship between the SNS, chronic stress, and fracture healing more comprehensively, particularly with regard to how chronic stress affects the nervous system’s involvement in the various stages of fracture healing. Understanding these interactions may lead to the development of more effective therapeutic strategies for fracture healing, potentially by targeting the SNS or stress response directly.

## Therapeutic Potential of Targeting Neural Pathways in Fracture Healing

Increasing our understanding of the neurobiology of fracture healing has opened up new avenues for potential therapeutic interventions focusing on the aspects of the nervous system involved in the healing process. One compelling approach involves modulating the activity of neurokinin-1 receptors (NK1; SP receptors). As mentioned above, poor fracture healing, as suggested by a smaller callus volume, decreased biomechanical strength of healing bone, and decreased angiogenesis, has been correlated with lower levels of SP in mice [57]. Conversely, evidence suggests that deliberate activation of NK1 expedites bone repair in a dose-dependent manner, potentially through positive effects on the inflammatory response and promotion of osteoblast proliferation [24, 71, 135]. Thus, SP agonists could be used to enhance fracture healing. One theoretical drawback to the use of SP agonists is the fact that there is evidence that SP has a positive modulatory effect on pain [66]. However, although behavioral evidence in animals suggests that NK1 antagonists decrease pain, studies in humans have found no analgesic effects of NK1 antagonists [136, 137]. Although data does not exist on the effects of NK1 agonists on pain in humans, the lack of impact of NK1 antagonism on pain decreases the concern for hyperalgesia from SP agonists. However, there are some additional drawbacks to use of SP, due to its link to neurogenic inflammation in animal models as well as the fact that its intradermal injection produces a wheal and pruritis in humans [138, 139].

Another tactic to accelerate fracture healing could involve modifying the levels of neurotrophic factors such as NGF and BDNF. Studies have demonstrated that strengthening BDNF signaling via administration of R13, a small molecule that interacts with BDNF’s receptor TrkB, results in improved bone repair in mice [140]. Moreover, applying a BDNF-containing paste to fill the gap created while inducing a femoral fracture increased bone formation and promoted fracture healing [141]. Additionally, administration of gambogic amide, an agonist of TrkA (NGF receptors), led to increased biomechanical strength as well as calluses with increased fractional bone volume 21 days after fracture [142•]. It is conceivable that the targeted manipulation of neurotrophic factors could optimize MSC recruitment and differentiation, enhancing fracture healing and tissue regeneration.

Due to the important role that NGF plays in the generation and maintenance of nociceptive pain, it is not surprising that experiments in mice demonstrated that administration of an anti-NGF monoclonal antibody diminished pain-related behaviors following fracture without negatively impacting callus bridging or the biomechanical strength of the healing femur [106, 107, 143]. A key concern with the use of these drugs in humans, though, has been reports of osteonecrosis, extensive bone damage, and rapidly progressing joint destruction leading to the necessity for joint replacement [144, 145]. These reports led to the US Food and Drug Administration [145] to vote decisively against approving tanezumab, an anti-NGF drug for osteoarthritis pain [146].

As the case of anti-NGF therapy has demonstrated, these promising neural targets come with inherent complexities. A primary concern is the interconnected nature of the peripheral nervous system and the risk for other physiological systems to be impacted when targeting aspects of the nervous system [147, 148]. Despite this challenge, the therapeutic potential of targeting neural pathways in fracture healing offers promising pathways for advancing bone repair and regeneration strategies.

## Conclusion

**Fig. 1:**
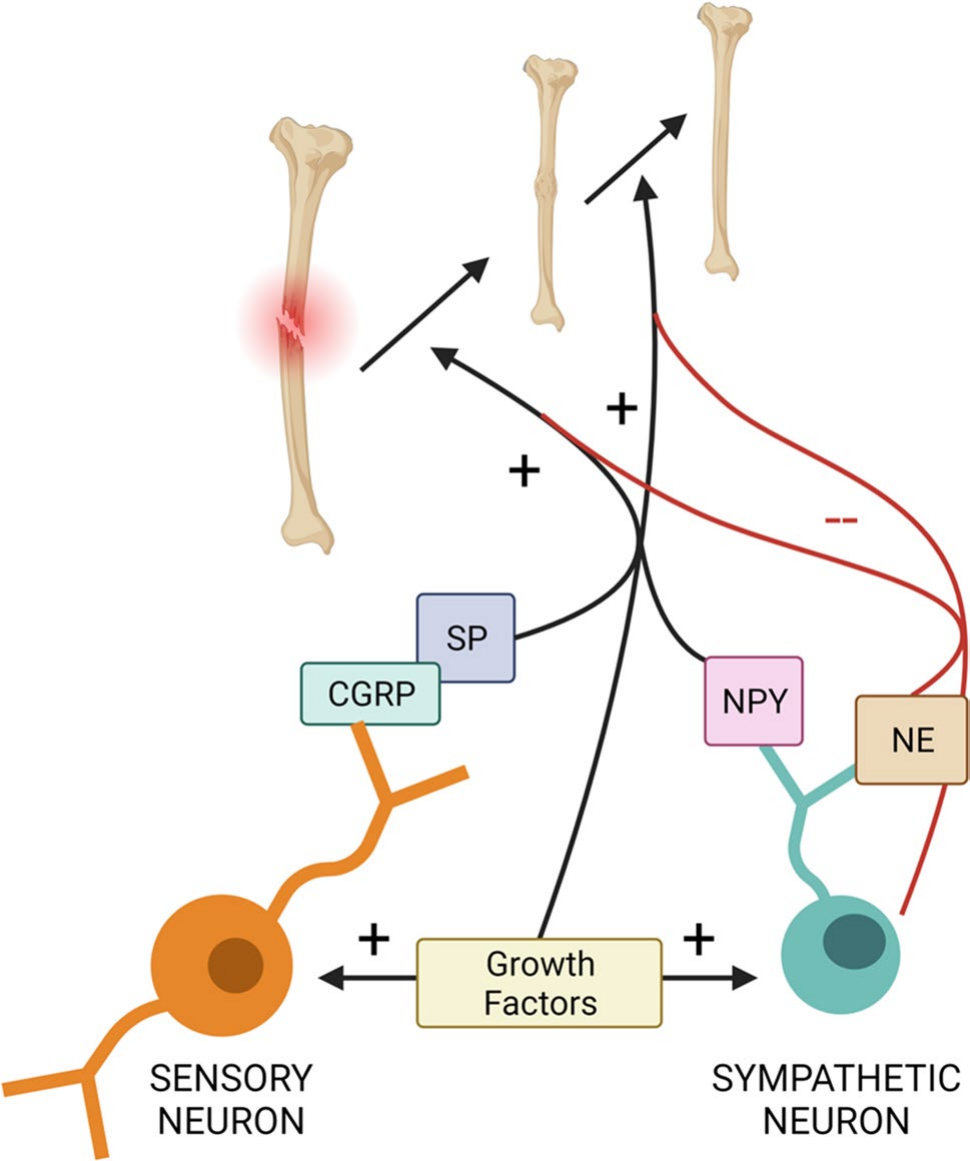
Nervous system regulation of fracture healing. This figure summarizes the contributions of the various aspects of the nervous system to fracture healing that were emphasized in this review. Growth factors and neuropeptides, including substance P (SP), calcitonin gene-related peptide (CGRP), and neuropeptide Y (NPY), all exert positive influences on fracture healing. The sympathetic nervous system, which releases norepinephrine (NE), can positively or negatively impact fracture healing depending on the degree of its involvement; local sympathectomies may improve fracture healing, while total systemic sympathetic denervation impairs fracture healing. Created with BioRender.com

The peripheral nervous system, as recent research has highlighted, plays a fundamental role in the physiological process of fracture healing, actively influencing a wide range of cellular and molecular events (Fig. 1). The impetus for deepening this understanding is twofold: scientific and clinical. The development of novel drugs targeting aspects of the nervous system could potentially enhance the speed and quality of fracture healing, alleviate complications associated with fractures such as pain, and decrease the burden on healthcare systems.
